# Clinical Characteristics of Children and Adolescents With Misophonia

**DOI:** 10.1177/00099228261467192

**Published:** 2026-09-19

**Authors:** Lotte R. Rappoldt, Carola de Wit, Marthe M. van der Pol, Simone Slaghekke, Caroline Houben, Kees J. Kan, André B. Rietman, Damiaan Denys, Elisabeth M. W. J. Utens, Nienke C. C. Vulink

**Affiliations:** 1 Department of Psychiatry, Amsterdam UMC, University of Amsterdam, The Netherlands; 2 Levvel, Academic Center for Child and Adolescent Psychiatry, Amsterdam, The Netherlands; 3 Research Institute of Child Development and Education, University of Amsterdam, The Netherlands; 4 Department of Child and Adolescent Psychiatry/Psychology, Erasmus MC Sophia Children’s Hospital, Rotterdam, The Netherlands

**Keywords:** misophonia, children, adolescents, quality of life, sensory processing

## Abstract

Misophonia has a serious impact on children and families. Few studies have, however, assessed the specific phenomenology of misophonia in youth. We investigated clinical characteristics in a treatment-seeking sample of youth with misophonia (n = 95, 8-18 years), using validated instruments to assess misophonia phenomenology, psychiatric comorbidity, emotional/behavioral problems, attention problems, sensory processing, and quality of life. The most prevalent triggers were eating sounds and the most common responses irritation/anger and anticipation anxiety. Psychiatric comorbidity was present in 27.4% of cases (eg, anxiety disorders 10.5%; attention-deficit hyperactivity disorder 8.4%). Internalizing psychopathology was more prevalent than externalizing. Adolescents reported differences in auditory, tactile, visual, and taste/smell sensory processing compared with a norm group, as well as heightened sensory awareness and avoidance. Misophonia severity was inversely related to quality of life in children/parents. Future research should investigate the underlying mechanisms of misophonia in youth. Pre-registered: The Netherlands Trial Register (CCMO): NL-OMON20775, https://onderzoekmetmensen.nl/nl/trial/20775

## Introduction

Misophonia is characterized by a decreased tolerance for specific sounds such as eating and nasal sounds and/or associated stimuli.^1^ These stimuli (“triggers”) induce immediate and strong feelings of irritation, anger, or disgust, along with adverse physiological and behavioral reactions.^2^ Triggers are contextual, since the same stimulus does not necessarily evoke similar strong reactions across situations. For instance, reactions might be stronger for close family members than for strangers.^3^ Confrontation with triggers is accompanied by a hyperfocus on the trigger, anticipatory anxiety, and/or avoidance.^2^

Misophonia was first described in the early 2000s^4,5^ and has since been conceptualized differently across different disciplines.^2,6-8^ In 2022, experts reached a consensus definition, and misophonia was identified as a disorder.^1^ Nevertheless, consensus regarding diagnostic criteria is still lacking and misophonia has neither been described in *the International Classification of Diseases*, 11th Revision *(ICD-11)*^9^ nor in the *Diagnostic and Statistical Manual of Mental Disorders*, Fifth Edition (*DSM*-5).^10^ Recent evidence points toward the categorization of misophonia as a distinct disorder^11^ that should be differentiated from other disorders such as hyperacusis (ie, decreased sound tolerance to subjectively experienced loudness of sounds).^7,12,13^

Misophonia typically develops in childhood or early adolescence and can have a serious impact on family relationships and quality of life.^14,15^ Knowledge of phenomenology of misophonia in youth is crucial for gaining insight in its etiology and the development of interventions. Previous research in two internet-based samples (Guzick et al:^14^ n = 102, Siepsiak et al:^16^ n = 45) and one clinical treatment-seeking sample (Lewin et al:^17^ n = 47) of youth with misophonia (aged between 7 and 18) found a relatively consistent clinical presentation of misophonia, with the most prevalent triggers including (human-made) eating, breathing, and throat sounds (eg, throat clearing), and common emotional responses such as annoyance/irritation, anger, distress, worry/anxiety, disgust, and frustration. Behavioral responses included verbal aggression (85%^14^; child-reported 57%, caregiver-reported 67%^17^) and occasional physical outbursts (50%^14^; child-reported 31%, caregiver-reported 33%^17^), which occurred more often in younger children (aged 7-12) compared with adolescents (aged 13-18) in the Siepsiak et al^16^ study. The majority of youth avoided specific family members and meals.^14,17^

Evidence of comorbidity is mixed. Both the Guzick et al^14^ study (n = 102) and the Lewin et al^17^ study (n = 47) reported comorbidity with social anxiety disorder (both 30%) and generalized anxiety disorder (27% and 36%, respectively). Comorbidity with major depressive disorder was higher in the study by Guzick et al^14^ (47% current or past, 15% current episode) than in the study by Lewin et al^17^ (8.5%). Interestingly, both of these studies found higher-than-expected comorbidity with attention-deficit hyperactivity disorder (ADHD) (21% and 15%, respectively); however, the Siepsiak et al study reported no differences in incidence of ADHD between children with misophonia (n = 45) and peers without misophonia (n = 38). Higher comorbidity with obsessive-compulsive disorder (OCD) was found in the 2 internet-based samples (eg, 8%^14^) compared with the clinical sample (2.1%).^17^ Elevated autistic traits were found in the Guzick et al^14^ study but not in the studies of Lewin et al^17^ and Siepsiak et al^16^ (in the latter, children with diagnosed autism spectrum disorder were excluded).

The comparability of these studies is complicated by the absence of unified diagnostic guidelines for misophonia. While all 3 studies incorporated either the Amsterdam UMC 2013^8^ or the Amsterdam UMC 2020^2^ proposed diagnostic criteria, the *exact* criteria for including children/adolescents with misophonia differed. In the internet-based study of Siepsiak et al,^16^ children with misophonia were identified based on a face-to-face interview with a psychologist (familiar with misophonia) using the 2020 criteria. The internet-based Guzick et al^14^ study also used a diagnostic interview (the Misophonia Assessment Interview [MAI],^18^ which is based on the 2013 criteria), but only included children/adolescents scoring ≥10 on the Amsterdam Misophonia Scale (A-MISO-S^8^). In contrast, the clinical sample of Lewin et al^17^ was identified by a clinician-rated severity score of ≥4 on the MAI and/or a clinician-rated misophonia severity score of ≥3 on the Clinical Global Impression—Severity Scale. Notably, these studies did not explicitly mention differentiation between misophonia and hyperacusis. To gain a deeper understanding of misophonia in youth, larger samples with validated assessment instruments are needed.

Previous studies hypothesized that difficulties in sensory processing might be a transdiagnostic factor contributing to misophonia phenomenology.^14^ Preliminary evidence can be found in the study of Armstrong et al,^19^ in which sensory sensitivity, measured by the total score on the Glasgow Sensory Questionnaire (GSQ),^20^ was associated with internalizing and externalizing symptoms in 102 youth with misophonia. Another study that used the parent-reported GSQ found that their sample of 13 children with misophonia displayed more *hyper*-sensitivity than 119 controls across all senses, as opposed to only in the auditory domain as can be expected with misophonia.^21^ However, the GSQ only provides insight in (affective/behavioral) *responses* to sensory stimuli, but not in *neurological thresholds* of youth with misophonia.^22^ To gain an understanding of sensory processing, it is important to assess the *interaction* between neurological thresholds, ranging from high to low thresholds, and behavioral responses, ranging from responding in accordance with thresholds to responding by counteracting thresholds (based on the conceptual model of Dunn, 1997).^23^ This interaction can be measured with the self-reported Adolescent/Adult Sensory Profile (AASP).^24^ Insight in sensory processing is essential for informing clinical practice and developing interventions that fit the sensory needs of children and adolescents. To the best of our knowledge, no studies have assessed sensory processing in youth with misophonia using the AASP.

This study aims to extend the current body of knowledge by including a large treatment-seeking sample of youth with misophonia (n = 95, aged 8-18), and investigating demographic characteristics, misophonia phenomenology, psychiatric comorbidity, emotional and behavioral problems, attention problems/ADHD characteristics, sensory processing, and quality of life. To assess misophonia phenomenology and severity, we used the recently validated (child- and parent-reported) Misophonia Screening List—Child and Youth and Amsterdam Misophonia Scale—Youth (AMISOS-Y), which displayed excellent psychometric properties.^25^ Since this research was exploratory, no a priori hypotheses were formulated.

## Materials and Methods

### Participants and Procedures

Ninety-five children/adolescents with misophonia were included (76.8% girls), with a mean age of 13.2 years (range = 8-17 years, SD = 2.4), as part of a randomized controlled trial (RCT) investigating the efficacy of group treatment (see Rappoldt et al^26^ for a detailed description of study procedures).

Participants from across the country were referred by general practitioners to our academic clinic for diagnosis and treatment for misophonia. Several procedures were undertaken before participants were included: file review, telephone screening, intake, and a diagnostic interview (see Figure 1). Because of the high number of referrals, we first *reviewed patient files* for the inclusion and exclusion criteria, after which we contacted participants for a *screening by telephone. Included* were children who (1) were aged between 8 and 18 years; (2) were referred by their general practitioner to our academic clinic for assessment and treatment of misophonia before January 2023; and (3) provided written informed consent (children 12+, together with both parents).

**Figure 1.**
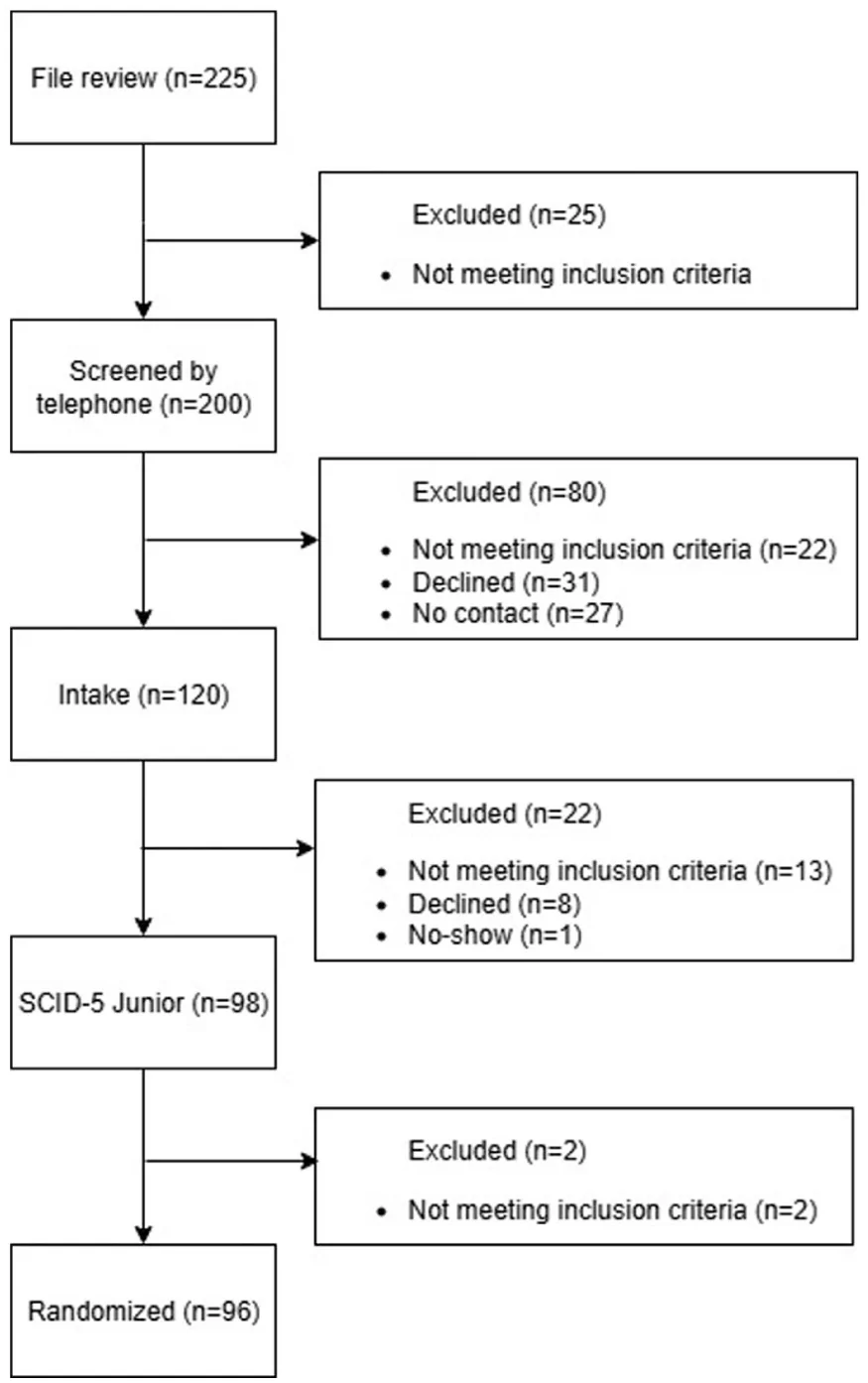
Inclusion procedure and reasons of exclusion. Since 1 randomized participant did not fill out any questionnaire, data of 95 participants were used.

*Excluded* were children who (1) presented primarily with symptoms or a diagnosis different from misophonia, meaning that misophonia symptoms were not the main problem during screening or intake (as mentioned by the child/parent or assessed during intake); (2) displayed comorbid psychiatric symptoms that hindered group functioning or required adjustment of treatment protocol (eg, if symptoms of autism spectrum disorder severely complicated group functioning for the child; as assessed during intake); (3) had received cognitive behavioral therapy for misophonia in the past year; (4) displayed self-injurious behavior (ie, auto-mutilation) at present or in the past year; (5) had an estimated IQ below 85 (as assessed during intake); (6) were unable to read or write Dutch; and (7) had serious family problems (eg, divorcing parents) that could hinder adjustment to treatment protocol (as assessed during intake), since parents were actively involved in treatment.

After file review and telephone screening, children and parents were invited for an *extensive intake* with trained psychiatrists/psychologists (with misophonia expertise) at our clinic. For this intake, we developed a standardized protocol (available upon request), which included questions about (not limited to): the clinical presentation of misophonia (eg, onset, course, emotional/behavioral/physical responses to triggers, coping, consequences), psychiatric comorbidity, attention/sensory processing problems, affect, previous psychological treatment, family history, and somatic symptoms. We also included screener questions for hearing problems (eg, Is there hearing impairment or hearing loss?), hyperacusis (eg, Are sounds often too loud for you, while others find them normal?), tinnitus (eg, Do you hear sounds in your ear(s) or head, that are not present around you?), and phonophobia (eg, Are you sometimes afraid to be in situations with certain sounds, because of your reaction to these sounds?). The presence of misophonia was determined using the Amsterdam UMC 2020 proposed diagnostic criteria,^2^ which reflect the international consensus definition of misophonia.^1^

Following intake, eligible potential participants were informed about the research verbally and in writing by the research team. Written informed consent was obtained from children (12+) and both parents after a minimum reflection period of 5 days. Then, the *semi-structured SCID-5 Junior diagnostic interview*^27^ was administered online (see also: Measures) to assess psychiatric comorbidity in detail, and as a final check of the inclusion and exclusion criteria. Afterwards, the participants were included (and randomized for the RCT^26^) and they completed the measurements of this study online as baseline assessment for the RCT. The RCT was approved by the Medical Ethics Committee AMC.

### Measures

*Demographic characteristics* were reported by parents using a short questionnaire. We included 2 variables as proxy measure of socioeconomic status (SES): parental highest completed education (trichotomized into low, intermediate, and high according to the Dutch classification system^28^) and parental occupation (categorized into low, low-intermediate, high-intermediate, and high based on the International Standard Classification of Occupations [ISCO]-08).^29^

*The phenomenology of misophonia* was assessed by the validated child- and parent-reported versions of the 11-item Misophonia Screening List—Child and Youth^25^ (range 0-44) and the 7 descriptive items (about triggers and emotional responses to triggers) and 10 Likert-scale items of the (child- and parent-reported) AMISOS-Y (range 0-40).^25^ Higher scores indicate more presence and higher severity of misophonia symptoms. Both questionnaires displayed good internal consistency in this study (child-report: α = .85, parent-report: .86; child-report: .79, parent-report: .83, respectively).

*Psychiatric comorbidity* was assessed by the Structured Clinical Interview for *DSM*-5 disorders in children and adolescents (SCID-5 Junior^27^). Due to Covid-19, this interview was conducted online. For children aged 8 to 12, the interview was administered at once (with child and parent(s) answering together); for children aged 13 to 18, the interview was administered with child and parent(s) separately. In total, 94.8% of SCID-5 Junior interviews were conducted by the first author (LR), supervised by principal investigators of this study (EU, NV, both licensed psychologists/psychiatrists). The remaining 5.2% of interviews were conducted by trained master’s students and checked by a second rater (LR).

*Emotional and behavioral problems* were assessed with the Child Behavior Checklist (CBCL; parent version, 120 problem items) and the Youth Self-Report (YSR; child version 11+, 105 problem items),^30,31^ of which we calculated the Total, Internalizing and Externalizing problem scales and the empirical subscales (ie, syndrome scales). Higher scores indicate more problems. Scores were compared with norm data to determine if they fell within the normal, borderline, or clinical range (in accordance with the manual^30,31^). Scores within borderline or clinical range indicated problems of (clinical) concern. *T*-scores between 60 and 63 were defined as borderline, and above 63 as clinical for the Total, Internalizing and Externalizing scales; *T*-scores between 65 and 69 were defined as borderline, and above 69 as clinical for the empirical subscales. In this study, the CBCL and YSR total problem scales displayed excellent internal consistency (resp. α = .94, α = .93).

*Attention problems and ADHD characteristics* were assessed with the 18-item Dutch ADHD questionnaire (in Dutch: ADHD-vragenlijst [AVL]^32^), which consists of a Total scale, and the subscales attention deficit, hyperactivity, and impulsivity. Higher scores indicate a higher symptom frequency. Total and subscale scores were compared with empirical norm data of the general youth population.^32^ Scores between the 90th and 95th percentiles were considered subclinical, scores of the 95th percentile and above clinical (as per manual). Scores within the subclinical or clinical range indicate problems requiring clinical attention. The AVL displayed excellent internal consistency in this study (α = .92).

*Sensory processing* was assessed with either the 38-item Short Sensory Profile—NL (SSP-NL)^33^ (if the child was between 8 and 10 years old, filled in by parents), or the 60-item Adolescent/Adult Sensory Profile—NL (AASP-NL)^24^ (self-reported if the child was 11 years or older). The SSP-NL provides an indication of sensory processing on seven domains: tactile sensitivity, taste/smell sensitivity, movement sensitivity, under-responsivity/seeking sensation, auditory filtering, low energy/weakness, visual/auditory sensitivity. *Lower* domain scores indicate behavior that is *more* consistent with the measured construct (eg, low scores on tactile sensitivity indicate more tactile sensitivity). Internal consistency was good (α = .80). Scores were compared with a norm group of the general youth population in the Netherlands.^33^

The AASP-NL integrates the interaction between neurological thresholds (high-low) and behavioral responses (passive = in accordance with thresholds – active = counteracting thresholds) in scores on 4 quadrants: low registration (high threshold, passive), sensation-seeking (high threshold, active), sensory sensitivity (low threshold, passive), sensation-avoiding (low threshold, active). Higher scores for low registration and sensation-seeking indicate *more* missing and seeking of sensory information. Higher scores for sensory sensitivity and sensation avoiding indicate a stronger response to (*more* sensitivity to) sensory information than the norm group. The 4 quadrants displayed good internal consistency in this study (α = .81, .72, .79, .80, respectively). In addition, domain scores were calculated by summing the items for each of the 6 domains (tactile, taste/smell, movement, activity, visual, and auditory), with *higher* domain scores indicating more behavior consistent with the measured construct. The AASP-NL scores were compared with norm groups of the general (youth) population.^24^

*Quality of life* was assessed with the 28-item Child Health Questionnaire (CHQ)-PF28 (parent version) and the 45-item CHQ-CF45 (child version, calculated according to CHQ-CF87 guidelines).^34^ Results were described in terms of scale scores (range 0-100), with higher scores indicating better health. Internal consistency was good (α = .87 and .85, respectively).

### Statistical Analysis

All statistical analyses were conducted using SPSS Statistics 28. Overall, the sample was reported descriptively in terms of means and standard deviations or percentage of the sample. For the SSP-NL (with limited sample size), one-sample Wilcoxon signed-rank tests were performed to compare our sample with the norm group. For the AASP-NL, we performed one-sample *t* tests. For the CHQ (PF-28 and CF-45), we used Pearson’s correlation (*r*) to assess the association between parent- and child-reported misophonia severity (on the AMISOS-Y). We only included participants who completed all relevant segments of the questionnaires. Interpretation of effect sizes was performed according to Cohen’s guidelines for *r* (0.10 = small effect; 0.30 = medium; 0.50 = large) and Cohen’s *d* (0.20 = small effect; 0.50 = medium; 0.80 = large).^35,36^

## Results

### Demographic Characteristics

Table 1 provides a summary of demographic characteristics of the sample. The majority of the sample had high SES (73.7% high parental completed education; 70.5% high parental occupational level).

**Table 1.** Demographic Characteristics of Clinical Sample (n = 95).

Characteristic	Total n (100%) = 95	SD or %
	M or n	
Age (M, SD)	13.2	2.4
Gender (n, %)^a^		
Girl	73	76.8%
Boy	22	23.2%
Country of birth (n, %)		
The Netherlands	91	95.8%
Other European country	1	1.1%
Non-European country	1	1.1%
Did not respond	2	2.1%
Divorced parents (n, %)	12	12.6%
Siblings (n, %)		
No siblings (only child)	6	6.3%
One sibling	55	57.9%
Two or more siblings	34	35.8%
Education (n, %)		
Primary	29	30.5%
Secondary^b^	66	69.5%
Parental highest completed education level (n, %)^c^		
Low	2	2.1%
Intermediate	23	24.2%
High	70	73.7%
Parental occupational level (n, %)^d^		
Low-intermediate	13	13.7%
High-intermediate	13	13.7%
High	67	70.5%
Not applicable/other	2	2.1%

Abbreviation: %, valid percentage.

a In our study, results of sex and gender (identity) were identical; therefore, only gender is reported.

b Including vocational education.

c Categorization according to Dutch classification system.^28^

d Categorization according to ISCO-08.^29^

### Phenomenology of Misophonia

Mean scores on the Misophonia Screening List—Child and Youth and AMISOS-Y are presented in Table 2. Almost all children (98.9%) reported to be triggered by eating sounds, such as chewing, lip smacking, slurping, and swallowing (Table 2). Other common triggers were nose and mouth sounds (89.4%; eg, blowing, sniffling, breathing, snoring, and coughing) and tapping sounds (64.9%; eg, nails on the table, clicking of a pen, a clock, and a keyboard; Table 2). The most prevalent emotional responses to triggers according to both children and parents were irritation, anger and anticipation anxiety (Table 2). Disgust was reported by a minority of children and parents (38.3% and 33.7%, respectively; Table 2).

**Table 2.** Misophonia Phenomenology in Clinical Sample (n = 95).

	Child-report (n = 94)		Parent-report (n = 95)	
Total scores (M, SD)				
Misophonia Screening List—Child and Youth	26.4	8.3	25.1	8.3
AMISOS-Y	22.4	5.7	22.4	5.6
Triggers (n, %)				
Eating sounds	93	98.9%	93	97.9%
Nose and mouth sounds	84	89.4%	82	86.3%
Voice sounds	40	42.6%	34	35.8%
Tapping sounds	61	64.9%	55	57.9%
Rustling sounds	36	38.3%	32	33.7%
Sounds from outside	15	16.0%	12	12.6%
Emotional responses (n, %)				
Irritation	91	96.8%	87	91.6%
Anger	80	84.0%	80	84.2%
Disgust	36	38.3%	32	33.7%
Afraid in advance (anticipation anxiety)	77	81.9%	69	72.6%
Sadness	57	60.6%	43	45.3%
Feeling bad	70	74.5%	57	60.0%
Guilt	60	63.8%	44	46.3%
Shame	49	52.1%	40	42.1%

### Psychiatric Comorbidity

Of the participants, 72.6% did not meet criteria for a comorbid diagnosis, 16.8% met criteria for 1 comorbid diagnosis, and 10.6% for 2 or more (Table 3). One or more comorbid anxiety disorder was found for 10.5% of participants, of which social anxiety disorder (3.2%) and specific phobias (3.2%) were the most common. Other comorbidities included ADHD (8.4%), tic disorders (7.4%), and insomnia disorder (6.3%).

**Table 3.** Overview of Psychiatric Comorbidity in Participants (SCID-5 Junior).

Comorbid diagnosis	Total n (100%) = 95	
	n	%
Comorbid diagnosis (total)	26	27.4
Attention-deficit hyperactivity disorder	8	8.4
Combined	2	2.1
Predominantly inattentive	6	6.3
Tic disorders	7	7.4
Tourette’s disorder	1	1.1
Persistent motor or vocal tic disorder (motor tics only)	5	5.3
Provisional tic disorder	1	1.1
Depressive mood disorders	4	4.2
Depressive disorder	3	3.2
Full remission	1	1.1
Partial remission	1	1.1
Persistent depressive disorder	1	1.1
Full remission	1	1.1
Disruptive mood dysregulation disorder	0	0.0
(Hypo)manic episode	0	0.0
Bipolar I/II disorder	0	0.0
Anxiety disorders	10	10.5
Social anxiety disorder	3	3.2
Specific phobia	3	3.2
Animal type	1	1.1
Natural type	1	1.1
Other types	1	1.1
Separation anxiety disorder	2	2.1
Generalized anxiety disorder	2	2.1
Selective mutism	0	0.0
Panic disorder	0	0.0
Agoraphobia	0	0.0
Obsessive-compulsive and related disorders	2	2.1
Obsessive-compulsive disorder	2	2.1
With good or fair insight	1	1.1
With absent insight/delusional beliefs	1	1.1
Body dysmorphic disorder^a^	0	0.0
Post-traumatic stress disorder	1	1.1
Illness anxiety disorder	0	0.0
Eating disorders	1	1.1
Avoidant/restrictive food intake disorder	1	1.1
Pica	0	0.0
Rumination disorder	0	0.0
Anorexia nervosa^a^	0	0.0
Bulimia nervosa	0	0.0
Binge eating disorder	0	0.0
Insomnia disorder	6	6.3
Disruptive, impulse control, and conduct disorders	1	1.1
Oppositional defiant disorder	1	1.1
Intermittent explosive disorder	0	0.0
Conduct disorder^a^	0	0.0
Substance use disorders^a^	0	0.0
Nonsuicidal self-injury disorder	0	0.0
Autism spectrum disorder	0	0.0
Other trauma and stressor-related disorders	0	0.0
Reactive attachment disorder	0	0.0
Disinhibited social engagement disorder	0	0.0
Brief psychotic disorder	0	0.0

Abbreviations: n, number of participants; %, valid percentage.

a Based on n = 94.

### Emotional and Behavioral Problems

In total, 36.6% of parents and 20.5% of children reported emotional and behavioral problems within the borderline and clinical range (Table 4). Clinical internalizing problems were reported by one third of parents (33.3%) and 17.9% of children. Of the clinical internalizing problems, parents most often reported somatic complaints (19.4%), and children anxious/depressed problems (6.4%). Clinical externalizing problems were reported relatively more often by parents (12.9%) than by children (1.3%), with parent-reported aggressive behavior being the most common (4.3%). Clinical attention problems were reported by 4.3% of parents and 11.5% of children.

**Table 4.** Borderline (Subclinical) and Clinical Emotional and Behavioral Problems in Participants.

Outcome	CBCL n (100%) = 93		YSR n (100%) = 78	
	Borderline^a^ n (%)	Clinical^a^ n (%)	Borderline^b^ n (%)	Clinical^b^ n (%)
Syndrome scales				
Internalizing	11 (11.8)	31 (33.3)	13 (16.7)	14 (17.9)
Anxious/depressed	19 (20.4)	8 (8.6)	4 (5.1)	5 (6.4)
Withdrawn/depressed	13 (14.0)	9 (9.7)	3 (3.8)	4 (5.1)
Somatic complaints	10 (10.8)	18 (19.4)	10 (12.8)	2 (2.6)
Externalizing	8 (8.6)	12 (12.9)	8 (10.3)	1 (1.3)
Rule-breaking behavior	4 (4.3)	0 (0)	1 (1.3)	0 (0)
Aggressive behavior	9 (9.7)	4 (4.3)	2 (2.6)	0 (0)
Social problems	5 (5.4)	2 (2.2)	3 (3.8)	5 (6.4)
Thought problems	11 (11.8)	5 (5.4)	3 (3.8)	1 (1.3)
Attention problems	14 (15.1)	4 (4.3)	5 (6.4)	9 (11.5)
Total problems	14 (15.1)	20 (21.5)	7 (9.0)	9 (11.5)

Abbreviations: n, number of participants; %, valid percentage.

a Compared with a US sample of 1753 parents.^30^

b Compared with a US sample of 1057 children (11-18 years old).^30^

### Attention Problems and ADHD Characteristics

(Sub)clinical total ADHD scores were found for 9.6% of children (Table 5). On the subscales, 14.9% of parents reported (sub)clinical attention-deficit problems, 8.5% hyperactivity problems, and 10.6% impulsivity problems.

**Table 5.** Parent-Reported Attention Problems and ADHD Characteristics in Participants.

AVL scale	Total n (100%) = 94	
	Subclinical n (%)^a^	Clinical n (%)^a^
Total ADHD	6 (6.4)	3 (3.2)
Attention deficit	4 (4.3)	10 (10.6)
Hyperactivity	5 (5.3)	3 (3.2)
Impulsivity	4 (4.3)	6 (6.4)

Abbreviations: n, number of participants; %, valid percentage.

a Compared with a general population norm group of 1480 children in the Netherlands (aged 4-18 years old).^32^

### Sensory Processing

On the SSP-NL, parents reported significantly higher scores than the norm group on 4 domains: taste/smell sensitivity, movement sensitivity, under-responsivity/seeking sensation, and visual/auditory sensitivity (large effect sizes, see Table 6). No difference was found on the other domains.

**Table 6.** Sensory Processing in Participants According to Parents (SSP-NL, n = 15).

SSP domain	Observed median (IQR)	One-sample Wilcoxon signed-rank test^a^	Effect size (*r*)
Tactile sensitivity	35 (2)	*Z* = 1.81, *P* = .071	
Taste/smell sensitivity	20 (2)	*Z* = 2.47, *P* = .014*	0.64
Movement sensitivity	15 (1)	*Z* = 3.16, *P* = .002*	0.82
Under-responsivity/seeking sensation	33 (2)	*Z* = 2.70, *P* = 007*	0.70
Auditory filtering	24 (9)	*Z* = −0.28, *P* = .776	
Low energy/weakness	30 (0)	*Z* = 1.09, *P* = .277	
Visual/auditory sensitivity	23 (3)	*Z* = 2.17, *P* = .030*	0.56

Abbreviation: n, number of participants.

a As compared with a norm group of 1257 normal developing children in the Netherlands (aged 6-10 years old).^33^

* Significant (*P* < .05, two tailed).

On the AASP-NL, children reported significantly *less* low registration and sensation seeking than to the norm group (small and large effect sizes, respectively), and *more* sensory sensitivity and sensation avoiding (small effect sizes, Table 7). With regard to the domains, children scored significantly lower on the taste/smell (large effect size, see Table 8), tactile and visual domain (small effect sizes), and significantly higher on the auditory domain (small effect size) than the norm group. No difference was found on the other domains.

**Table 7.** Sensory Processing in Participants According to Adolescents (AASP-NL quadrants, n = 77).

AASP quadrant	Mean (SD)	*z*-test^a^	One-sample *t* test^a^ (n = 77)	Effect size (Cohen’s *d*)
Low registration	29.9 (7.92)	−0.48	*t*(76) = −4.11, *P* < .001**	−0.47
Sensation seeking	39.6 (8.03)	−1.10	*t*(76) = −10.79, *P* < .001**	−1.23
Sensory sensitivity	36.1 (9.44)	0.29	*t*(76) = 2.01, *P* = .048*	0.23
Sensation avoiding	35.5 (8.95)	0.34	*t*(76) = 2.39, *P* = .020*	0.27

Abbreviation: n, number of participants.

a As compared with a norm group of 193 normal developing American adolescents (aged 11-17 years old).^24^

* Significant (*P* < .05, two tailed). **Significant (*P* < .001, two tailed).

**Table 8.** Sensory Processing in Participants According to Adolescents (AASP-NL domains, n = 77)

AASP domain	Mean (SD)	*z*-test^a^	One-sample *t* test^a^	Effect size (Cohen’s *d*)
Tactile	28.4 (6.8)	−0.53	*t*(76) = −3.70, *P* < .001**	−0.42
Taste/smell	16.8 (4.3)	−1.42	*t*(76) = −10.38, *P* < .001**	−1.18
Movement	19.3 (4.9)	0.10	*t*(76) = 0.65, *P* = .521	
Activity	25.2 (6.2)	−0.26	*t*(76) = −1.63, *P* = .107	
Visual	21.9 (5.1)	−0.48	*t*(76) = −3.70, *P* < .001**	−0.42
Auditory	29.5 (7.6)	0.48	*t*(76) = 3.25, *P* = .002*	0.37

Abbreviation: n, number of participants.

a As compared with a norm group of 959 American adolescents and adults of 11 years and older.

* Significant (*P* < .05, two tailed). **Significant (*P* < .001, two tailed).

### Quality of Life

Misophonia severity was inversely related to parent- and child-reported quality of life on several domains (Table 9). For parents, moderate negative associations were found between misophonia severity and self-esteem of the child and family activities. Furthermore, misophonia severity was moderately associated with parental emotional impact. For children, we found a moderate inverse relationship between misophonia severity and family activities, emotional/behavioral limitations, perceptions of general health, and mental health (Table 9). We found no association between misophonia severity and family cohesion.

**Table 9.** Pearson Correlation Between Quality of Life as Reported by Parents (CHQ-PF28) and Children/Adolescents (CHQ-CF45) and Misophonia Severity (AMISOS-Y).

CHQ scale	CHQ-PF28 (n = 94)			CHQ-CF45 (n = 92)		
	Mean (SD)	Correlation coefficient^a^	*P* value	Mean (SD)	Correlation coefficient^b^	*P* value
Global health	75.7 (20.2)	−.28*	.006	73.6 (19.4)	−.23*	.026
Physical functioning	95.5 (11.4)	−.28*	.007	92.5 (10.6)	−.26*	.012
Role/social limitations—emotional/behavioral	94.0 (14.6)	−.05	.637	82.1 (23.3)	−.37**	<.001
Role/social limitations—physical	95.7 (15.6)	−.05	.669	91.5 (16.9)	−.16	.132
Bodily pain/discomfort	75.1 (22.3)	−.09	.380	75.0 (24.3)	−.18	.084
Behavior	73.0 (15.2)	−.18	.079	72.8 (13.4)	−.06	.579
Global behavior item	71.2 (18.8)	−.08	.483	69.1 (18.4)	.07	.519
Mental health	75.0 (16.7)	−.24*	.020	69.0 (15.1)	−.32*	.002
Self-esteem	72.1 (15.8)	−.32*	.002	73.7 (13.7)	−.17	.100
General health perceptions	83.8 (18.6)	−.26*	.011	78.0 (17.4)	−.38**	<.001
Change in health	71.3 % about the same health now as 1 year ago			58.7% about the same health now as 1 year ago		
Parental impact—emotional	77.7 (20.2)	−.30*	.004	—	—	—
Parental impact—time	91.0 (17.2)	−.26*	.013	—	—	—
Family activities	59.1 (25.1)	−.30*	.003	74.8 (19.4)	−.43**	<.001
Family cohesion	62.8 (22.1)	−.05	.641	64.4 (23.2)	.10	.333

Abbreviations: n, number of participants; SD, standard deviation.

a df = 92.

b df = 90.

* *P* < .05 (two tailed). **P < .001 (two tailed).

## Discussion

This study presents clinical characteristics of the largest sample thus far of youth seeking treatment specifically for misophonia (n = 95, aged 8-18 years). Human-made eating, nose, and mouth sounds were the most prevalent triggers, which is consistent with previous research in youth.^14,17^ Furthermore, similar to previous research,^14,16,17^ irritation, anger and (anticipation) anxiety were the most pronounced emotional responses to triggers. However, while disgust is also considered a characteristic misophonic response^1,2^ that was often reported by children/adolescents in previous studies (eg, 68% in the study of Guzick et al^14^; 52.2% in the study of Lewin et al^17^), this was reported by a mere one third (38.3%) of our sample. When investigating emotions, such as disgust, it is important to account for social and cultural context.^37^ To illustrate, in misophonia, the same behavior (eg, lip smacking) might elicit disgust in some social situations (eg, performed by parents), but not in other situations (eg, performed by babies or elderly people). Similarly, the same behavior might be perceived differently across cultures, and might elicit disgust more readily in some cultures than in others. Furthermore, it is important to consider linguistic diversity. The Dutch word for “disgust,” which presumably has a more specific and negative connotation than the English word, is not necessarily part of standard emotion education in the Netherlands^38^ and might be less familiar to Dutch (younger) children than to English children, resulting in differential outcomes. This underlines the importance of social and cultural sensitivity in the interpretation of study results. In the future, qualitative research (see eg, Guzick et al^39^) might promote a deeper understanding of misophonia phenomenology across different socio-cultural contexts.

In this study, the majority (72.6%) did not display psychiatric comorbidity, which is in contrast to the treatment-seeking sample of Lewin et al^17^ (n = 47), in which a high incidence of comorbid diagnoses was found using a similar *DSM*-5 interview (ie, ADIS-5). Most notably, we found lower rates of anxiety disorders (10.5% compared with 59.6% in the study of Lewin et al^17^), including less social anxiety disorder (3.2% compared with 29.8% in the study of Lewin et al^17^) and generalized anxiety disorder (2.1% compared with 36.2% in the study of Lewin et al^17^). We also found lower incidence of depressive disorder (3.2% compared with 8.5% in the study of Lewin et al^17^). In our study, 8.4% of participants met criteria for ADHD (compared with 14.9% in the study of Lewin et al^17^), with attention deficit being the most elevated compared with a norm group. The incidence of tic disorders and OCD in our group was similar to the Lewin et al study (7.4% compared with 6.4% in the study of Lewin et al^17^ and 2.1% in both studies, respectively). It should be noted that in our study, participants were included for an RCT investigating group (CBT + PMT) treatment, and were excluded in case comorbid symptoms or diagnoses complicated or hindered group functioning (in which case individual treatment was indicated). In practice, this mainly concerned complex psychopathology and/or symptomatology of ADHD and autism spectrum disorder. Therefore, our more stringent inclusion procedure could be an explanation for the lower rates of comorbidity found in our study. However, our results are informative and worthwhile for clinical practice. Notably, we would recommend a similar inclusion procedure when starting and implementing group treatment. To illustrate, starting group treatment can be too demanding for some youth with autism spectrum disorder or severe OCD in addition to misophonia, and/or can influence group functioning. More research in diverse samples is necessary to inform the development of unified diagnostic guidelines, in which misophonia should be differentiated from disorders with similar symptom presentation (eg, social anxiety disorder, hyperacusis). In this regard, future studies should consider a multidisciplinary approach (see, eg, Chan et al^40^).

To identify to which cluster of diagnostic categories (eg, in *DSM*-5; internalizing disorders, somatic disorders, externalizing disorders^41^) misophonia would belong, it is insightful to assess patterns of internalizing and externalizing problems in youth with misophonia. In our study, we found a relatively higher percentage of clinical (parent- and child-reported) internalizing problems than externalizing problems, which is consistent with previous studies.^14^ Moreover, in a previous study of our sample, a significant positive correlation was found between (parent- and child-reported) misophonia severity and internalizing problems (*r* = 0.31 and 0.38, respectively), but no correlation with externalizing problems.^25^ This might point toward categorizing misophonia among the internalizing disorders. Interestingly, although verbal or physical aggression in response to triggers is commonly reported by youth with misophonia, this is not reflected in our findings on externalizing problems (ie, rule-breaking or aggressive behavior). In this light, we hypothesize that misophonia might not necessarily reflect difficulties in impulse control or disinhibition, but could rather be associated with *high* inhibition of emotions. If a child inhibits or suppresses their emotions during daily life for too long, their general stress levels might increase. One trigger (eg, eating sound), especially when produced within a surrounding in which the child feels safe (eg, with loved ones), might then induce an intense emotional outburst. The conflict between feeling these extreme negative emotions toward loved ones after being triggered, and wanting to refrain from acting on these emotions, might contribute to misophonia symptomatology.

Previous studies hypothesized that dysregulated sensory processing might be an important underlying transdiagnostic factor implied in misophonia in youth.^14^ In our study, for the adolescents (aged 11+, n = 77) the self-reported AASP was used to assess the interaction between neurological thresholds (high-low) and behavioral responses (passive-active) to stimuli in daily life. Compared with the norm group, adolescents reported a general pattern of *decreased* low registration and sensation seeking (both high thresholds; passive and active responses), and *increased* sensory sensitivity and sensation avoiding (both low thresholds; passive and active responses). First, *decreased* low registration and sensation seeking indicates *less* missing and seeking of sensory information, which implies that generally, the current environment might be sufficient in creating activation for adolescents with misophonia. They might refrain from seeking more (intense) sensory stimuli to prevent feeling overwhelmed. Second, *increased* sensory sensitivity and sensation avoiding corresponds with misophonia pathology, in which individuals display a hyper-awareness of specific sounds, and aim to avoid or reduce these sounds to function. Notably, we found differences from the norm group not only in the auditory domain (as could be expected in misophonia), but also in the tactile, taste/smell, and visual domains, which offers support for the hypothesis that general sensory dysregulation could be a transdiagnostic vulnerability. We found no evidence for differential sensory processing related to movement (eg, difficulties in balance, clumsiness) nor for activity level in daily life. In clinical practice, it is important to assess individually with children/adolescents how sensory processing patterns impact their daily life, and to recognize the possibility of additional sensory problems.

Interestingly, for the younger children in our sample (aged 8-10, parent-reported on the SSP-NL, n = 15), we found significantly *higher* scores (indicating *lower* sensitivity) on the taste/smell, movement and visual/auditory domain, and no difference for auditory filtering and tactile sensitivity. Possibly, differences in sensory processing might become more pronounced with age, for instance during puberty. Consistent with our findings in adolescents, we found less under-responsivity and sensation seeking behavior in younger children, and no differences in general energy level. Importantly, these SSP-NL results should be interpreted with caution due to the small number of younger children in our sample (n = 15).

In line with previous findings,^14^ we found that severity of misophonia was associated with reduced quality of life with regard to general health (compared with peers), physical functioning, mental health, and self-esteem. Furthermore, we found that misophonia was related to impaired family activities, and was associated with emotional impact (worry and distress) for parents. Misophonia is commonly centered around eating situations, for instance, at the dinner table, which might induce stress or tension in all family members. In this regard, it might be important to assess family accommodation, which refers to the way in which parents and other family members participate in certain behaviors to minimize the distress resulting from a disorder, for instance, by providing reassurance, or by modifying behaviors or routines.^42,43^ In a sample of 102 youth with misophonia, Storch et al^44^ found that family accommodation was common and was associated with misophonia severity. Future research should delineate to which extent family accommodation could be adaptive (eg, in reducing stress), or could be related to non-adaptive outcomes (eg, increased avoidance) in misophonia. Even though misophonia could be an important stressor for all family members, we found no evidence for impaired family cohesion in our study.

This study presents the clinical characteristics of the largest sample seeking treatment for misophonia thus far, using specific validated child- and parent-reported questionnaires to assess misophonia. The results of this study should be interpreted considering several limitations. First, our sample consisted mostly of girls from high SES families (similar to previous research), and we used a relatively stringent inclusion procedure; therefore, the generalizability of our results to the general population of youth with misophonia is limited. Second, since the misophonia assessment instruments were validated during the course of this study,^25^ we did not use cut-offs scores from these instruments to identify misophonia in our sample (as part of inclusion and exclusion criteria). The validated instruments were, however, used throughout the assessments in our sample to describe misophonia phenomenology and to perform correlative analyses. Third, although visual triggers, behavioral/physical responses to triggers and comorbid hearing problems, hyperacusis, tinnitus, and phonophobia were evaluated during the intake, these variables were not systematically assessed with questionnaires or other assessment instruments. Including complete and systematic assessment of these factors would have would have provided additional insight into misophonia phenomenology. Fourth, the majority of the semi-structured diagnostic interviews (SCID-5 Junior) to assess comorbidity was performed by one rater (LR). This rater was thoroughly trained and supervised by two licensed and expert psychiatrists/psychologists. In case of doubts, these were discussed until consensus was reached. Including a second rater and assessing interrater reliability would have further strengthened our outcomes.

In short, the findings of this study, performed in 95 Dutch treatment-seeking youth with misophonia and their parents, underscore that misophonia is a disorder associated with significant consequences for daily life of children and families. Misophonia primarily co-occurred with internalizing problems. In adolescents, we found evidence of dysregulated sensory processing across multiple senses, with a pattern of heightened awareness of sensory stimuli and increased sensory avoiding. We recommend that these novel findings on sensory processing are replicated in future studies. Furthermore, future studies should assess predisposing, precipitating and perpetuating factors of misophonia in youth in more detail, while remaining sensitive for social and cultural context.

## Author Contribution

LR, KJK, DD, EU, NV contributed to the study conceptualization and methodology. LR contributed to investigation. LR, KJK, AR contributed to data analysis and interpretation. DD, EU, NV provided supervision. LR drafted the manuscript. All authors reviewed the manuscript critically and approved the final version.

## Data Availability

Data are available upon reasonable request. Considering the fact that we have gathered highly sensitive and privacy protected data (protection legislated by Dutch law), we can only share relevant supporting documents in case of relevant research questions.
